# Characterization, Antimicrobial Properties and Coatings Application of Gellan Gum Oxidized with Hydrogen Peroxide

**DOI:** 10.3390/foods8010031

**Published:** 2019-01-17

**Authors:** Yushuang Lu, Xiaojian Zhao, Sheng Fang

**Affiliations:** School of Food Science and Biotechnology, Zhejiang Gongshang University, Xuezheng Street No. 18, Hangzhou 310018, China; lyszjgsl@163.com (Y.L.); zhaoxj0822@foxmail.com (X.Z.)

**Keywords:** oxidation, gellan gum, spectroscopy, gelation, antimicrobial

## Abstract

The effect of hydrogen peroxide (H_2_O_2_) oxidation on the physicochemical, gelation and antimicrobial properties of gellan gum was studied. The oxidized gellan gum (OGG) was characterized by measuring the carboxyl/carbonyl group contents, Fourier transform infrared spectroscopy (FTIR) and proton nuclear magnetic resonance (^1^H-NMR) spectroscopy. The H_2_O_2_ oxidation resulted in a large increase in the carboxyl groups in gellan gum. The OGG lost gelation ability by oxidation even in the presence of metal ions. The antimicrobial activities of the OGG against Gram-positive bacteria (*Staphylococcus aureus*), Gram-negative bacteria (*Escherichia coli*), and fungal (*Aspergillus niger*) were tested. The OGG could inhibit the growth of both bacteria and fungal, and the activity was improved with an increase in the oxidation level. Finally, the application of the OGG as an active coatings material to extend the storage of apples was tested.

## 1. Introduction

Microbial exopolysaccharides can be produced on a very large scale and with potential applications in many areas as renewable non-petrochemical-based materials. Application areas of exopolysaccharides are widening, ranging between foods, cosmetics, biomedical and daily-life products [1,2,3]. The chemical modifications of microbial exopolysaccharides can introduce hydrophobic, acidic, basic, or other desired functional groups into structures by esterification, etherification, sulfurization, amidation, and oxidation et al. which greatly extends their applications [4,5]. 

Gellan gum, as a commercially important *Sphingomonas elodea* exopolysaccharide, is a linear anionic polymer with tetrasaccharide repeats including one glucuronic acid, one rhamnose, and two glucose carbohydrates [6,7]. It has been well used in food, cosmetics, and bioengineering due to its acid and heat resistance, and good processing ability [7,8]. The physicochemical modification of gellan gum facilitated their applications in different areas. For example, gellan gum was carboxymethylated by reacting with monochloroacetic acid which gave them higher mucoadhesive strength [9]. Matricardi et al. [10] prepared a crosslinked gellan gum and showed good control release properties for drugs. A recent review [8] has reviewed the functionalization strategies of the gellan gum for biomedical applications. 

Oxidation has been widely used for the functionalization of gellan gums [8]. Redouan et al. [11] selectively oxidized the gellan gum by using sodium hypochlorite in the presence of 2,2,6,6-tetramethylpiperidine-1-oxyl radical (TEMPO) and NaBr in aqueous solution. They obtained a water-soluble natural polymer which might find applications in medical and pharmaceutical areas. Gong et al. [12] developed a hydrogel-based scaffolding system by oxidative cleavage on the gellan gum backbones with NaIO_4_ while providing a promising platform for cartilaginous regeneration. Tang et al. [13] used NaIO_4_ to oxidize the adjacent hydroxyl groups in the gellan gum, which resulted in gels with low gelation temperature and improved cell compatibility. Oxidant agents such as TEMPO, NaBr/NaOCl, N_2_O_4_, and H_2_O_2_ are always selected for the chemical modification of polysaccharides [14,15]. H_2_O_2_ has strong oxidizing properties and only releases water as a by-product [15]. H_2_O_2_ can oxidize the primary alcohol group to an aldehyde group, or further to a carboxyl group. The process can effectively introduce carboxyl and/or carbonyl groups into the treated polysaccharides [15]. The oxidation of the gellan gum by H_2_O_2_ has not been studied. More data about the effects of H_2_O_2_ oxidation on the physicochemical and functional properties of the gellan gum is essential for extending its applications. 

This study used H_2_O_2_ as a green oxidant to oxidize the gellan gum with copper sulfate as a catalyst. The structure and properties of the oxidized gellan gum (OGG) were characterized by determining the carbonyl/carboxyl content using Fourier transform infrared spectroscopy (FTIR) and the proton nuclear magnetic resonance (^1^H-NMR) spectrum. The gel properties of the OGGs were tested. In addition, the antimicrobial activities of the OGG against Gram-positive bacteria (*Staphylococcus aureus*), Gram-negative bacteria (*Escherichia coli*) and fungal (*Aspergillus niger*) were studied. To the best of our knowledge, the antibacterial activity of the OGG had not been tested. Finally, as an application, the OGG was tested as an antibacterial film material on food surfaces in the extending of shelf life. 

## 2. Materials and Methods 

### 2.1. Materials

The low acyl gellan gum was supplied by CP Kelco (San Diego, CA, USA). The low acyl gellan gum was checked by FTIR and ^1^H-NMR for structure, and gel permeation chromatography (GPC) for molecular weight. The H_2_O_2_ (30% in aqueous) was purchased from Sinopharm Chemical Reagent Co., Ltd. (Shanghai, China). *Escherichia coli* (*E. coli*, CGMCC1.8745), *Staphylococcus aureus* (*S. aureus*, CGMCC 1.1861) and *Aspergillus niger* (*A. niger* CGMCC3.3928) were obtained from China General Microbiological Culture Collection Center (CGMCC, Beijing, China). 

### 2.2. Preparation of Oxidized Gellan Gum

The OGG was prepared following the method of oxidized amyloses reported by Ying et al [16]. Briefly, 2 g of gellan gum was dissolved in 70 mL of deionized water at 90 °C. After the solution cooled to 50 °C, 4 mL of 0.05% CuSO_4_ solution was added, 30% H_2_O_2_ was then added at different dosages (10, 20, 30 and 40 mL) with stirring. The solution was allowed to react for 5 h. After the reaction, the mixture was dialyzed to remove Cu^2+^ and H_2_O_2_ before freeze drying. The process was checked by determining the conductivity of the dialysate. The oxidized gellan gums were denoted as OGG-1, OGG-2, OGG-3 and OGG-4 when the dosages of H_2_O_2_ were 10, 20, 30 and 40 mL, respectively. 

### 2.3. Carboxyl Content Determination

The carboxyl content was determined according to the calcium-acetate method [17]. The samples were kept in a vacuum oven at 60 °C for 48 h. 45 mg of selected OGG was dissolved in 30 mL water. Then 10 mL of 0.1 M calcium acetate was added. The mixture was stirred for 1 h to promote the reaction. Then, the liquid was titrated using 0.01 M NaOH. A blank determination with gellan gum was performed in the same manner. The carboxyl content was calculated as follows:

(1) $$\mathrm{COOH}(\%)=\frac{\left[V_{\mathrm{NaOH}}-V_{0}\right]\times 0.01\times 45\times 100}{m}$$

where *V*_NaOH_ and *V*_0_ was the volume of NaOH solution in titration for the OGG and control sample, respectively; *m* was the weight (g) of the OGG sample, and 45 was the molecular weight of the carboxyl group. 

### 2.4. Carbonyl Content Determination

The OGGs were converted to oxime by Schiff’s base reaction with hydroxylamine hydrochloride. The calibration curve was determined at first. Briefly, in different volumes (0.5, 1.0, 1.5, 2.0, 2.5, and 3.0 mL) of 4.22 × 10^−4^ M glyoxal solution, 1 mL of 1.5% (w/w) sodium acetate and 1 mL of 0.2% (w/w) hydroxylamine hydrochloride were added. The reaction was carried out at 50 °C for 20 min. Then the solution was diluted to 50 mL and measured at 233 nm using a UV-2600 spectrophotometer (Shimadzu, Kyoto, Japan). The calibration curve between the absorbance and carbonyl content was calculated as *y* = 9488.8*x* + 0.0085 (*x* is the carbonyl content, *y* is the UV absorbance). The OGG sample (10 mg) was dissolved in deionized water and measured. The carbonyl content of the OGGs was calculated as:

(2) $$\mathrm{CO}(\%)=\frac{(y-0.0085)\times 0.05\times 28\times 100}{9488.8\times m}$$

where *m* was the weight (g) of the sample and 28 was the molecular weight of the carbonyl group. Each set of tests was done in triplicate, and the average values were selected. 

### 2.5. Fourier Transform Infrared Spectroscopy (FTIR)

The FTIR spectra of the OGGs were obtained from a Fourier Transform Infrared Spectrometer (Nicolet is5, Thermo Scientific, Waltham, MA, USA). The samples were kept in a vacuum oven at 60 °C for 48 h. 1 mg of sample and 100 mg of KBr were mixed, ground, compressed and then tested. The range of wavenumbers was from 4000 cm^−1^ to 400 cm^−1^ with the resolution of 4 cm^−1^. 

### 2.6. Proton Nuclear Magnetic Resonance (^1^H-NMR) Spectroscopy

The proton nuclear magnetic resonance (^1^H-NMR) spectra of the OGGs were collected at 308 K by a Bruker Avance III 600 MHz NMR Spectrometer (Billerica, MA, USA). The unoxidized low acyl gellan gum needs to be dissolved at high temperatures, so the H-NMR spectra of the low acyl gellan gum was performed by an Avance III 400 MHz NMR Spectrometer (Billerica, MA, USA) at 353 K. The samples were dissolved in D_2_O at about 20 mg/mL. 

### 2.7. Gel Properties of the Oxidized Gellan Gum in the Presence of Ions

The OGG powder was dispersed in 100 mL deionized water at room temperature. It was then placed in a 90 °C water bath and stirring was undertaken until the powder was completely dissolved to form a transparent, clear solution. Then, 0.06% of CaCl_2_ or 1.7% of NaCl were added to the solution and stirred well until completely dissolved. Finally, it was left to cool naturally and its gel formation was observed. 

### 2.8. Antibacterial Activity of the Oxidized Gellan Gum

Two food-related bacterial strains *E. coli* and *S. aureus* were selected to evaluate the antibacterial property of the OGGs. The strain was inoculated in nutrient broth (NB), and then incubated at 37 °C for 12 h. The stationary phase was determined by optical density at 600 nm. A working solution was prepared by diluting the above subculture into PBS, with the final bacterial concentration of 10^6^ CFU (colony forming unit)/mL. The number of bacteria was measured by the plate counting method, which was performed three times to obtain representative CFU values. 

The minimum inhibitory concentration (MIC) was measured using a broth dilution method for bacteria. 1 mL of NB broth with various contents of the OGG was placed into a 24-well plate. Then 20 μL of bacterial suspension was added to each well and incubated for 24 h with constant shaking at 150 rpm and 37 °C. The minimum concentration of the sample at which microbial growth was measurably inhibited was taken as the MIC. 

### 2.9. Antifungal Activity of the Oxidized Gellan Gum

The antifungal activity of the OGG was measured by evaluating the ability to control *A. niger* growth according to Reference [18]. 1 mL of potato dextrose broth (PDB) containing various contents of the OGG was placed into each well of a 24-well plate. Then 100 μL of *A. niger* suspension was added to each well. The culture medium was incubated at 28 °C for 24 h. After that, 100 μL of culture medium was collected and reincubated on potato dextrose agar (PDA) plates at 28 °C for 2 days. The appearances of *A. niger* colonies on PDA plates were observed to evaluate the antifungal activity of the OGG.

### 2.10. Effect of the Oxidized Gellan Gum on Apple Preservation

Apples were selected with similar maturity, size, and shape. Then washed and disinfected in sodium hypochlorite with a concentration of 0.05% (v/v). Cut the same incision on each apple surface and apply the prepared coating-forming solution separately. Three groups of coating-forming solution were prepared: (A) 5 mg/mL guar gum and 10 mg/mL glycerol; (B) 5 mg/mL guar gum, 10 mg/mL glycerol and 10 mg/mL OGG-4; (C) 5 mg/mL guar gum, 10 mg/mL glycerol and 20 mg/mL OGG-4. The appearance of the apple was recorded at different times by taking photos. 

## 3. Results and Discussions

### 3.1. Characterizations

#### 3.1.1. Carboxyl and Carbonyl Contents of the Oxidized Gellan Gums

Table 1 showed the carboxyl and carbonyl contents of different OGGs. It showed that the increase in the H_2_O_2_ amount resulted in a corresponding increase in the carbonyl and carboxyl contents. The carbonyl content increased from 0.36% to 0.87%, while the carboxyl content increased from 6.11% to 14.51% and the H_2_O_2_ increased from 10 to 40 mL. The carbonyl content is much less than the carboxyl content. The same behavior in oat *β*-glucan was observed by Moura et al. [19], that the carbonyl and carboxyl contents increased with increasing H_2_O_2_ level and reaction time. These results suggest that the OGG with different oxidation levels are successfully prepared and the carbonyl and carboxyl content can be adjusted. 

#### 3.1.2. Fourier Transform Infrared Spectroscopy Analysis 

**Figure 1:**
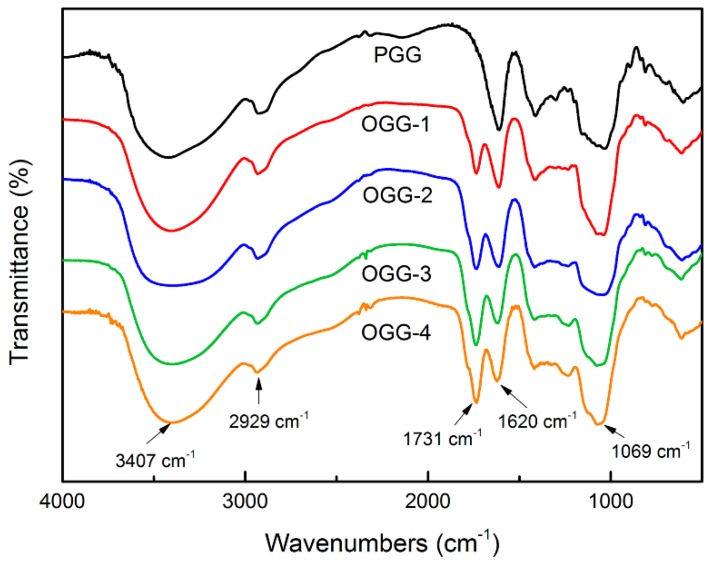
FTIR (Fourier transform infrared spectroscopy) spectra of pure gellan gum (PGG) and oxidized gellan gum (OGG) with different degrees of oxidation.

Figure 1 shows that the pure gellan gum (PGG) and all the OGGs present characteristic peaks at around 2929, 1620 and 1069 cm^−1^. The band corresponding to C-H vibrations of sugar occurs at approximately 2929 cm^−1^. The strong band at around 1069 cm^−1^ was attributed to the C-O-C stretching. The result showed that all the OGGs had a new characteristic peak around 1731 cm^−1^. The peaks became more significant with the increase of oxidation degree. Similar results reported that new absorption peaks around 1730 cm^−1^ were detected in the oxidized konjac glucomannan by H_2_O_2_ [20]. It is known that esters, aldehydes, and carboxylic acids will have C=O stretching peaks around 1715 cm^−1^. The carboxylic group exhibits a peak around 1730 cm^−1^ in the dissociated form, and 1620 cm^−1^ in the salt form [21]. So, the increasing of band peak around 1731 cm^−1^ indicates the formation of carboxylic groups in the OGGs which is in accordance with the findings in Table 1. Similar results were observed in oxidized amylose and oxidized cellulose when the hydroxyl groups were transformed to carbonyl and carboxyl groups [22]. These results showed that the carboxyl groups had been successfully introduced into the structure of gellan gum by H_2_O_2_ oxidation and supported the proposed oxidation mechanisms. 

#### 3.1.3. Nuclear Magnetic Resonance Spectrum

**Figure 2:**
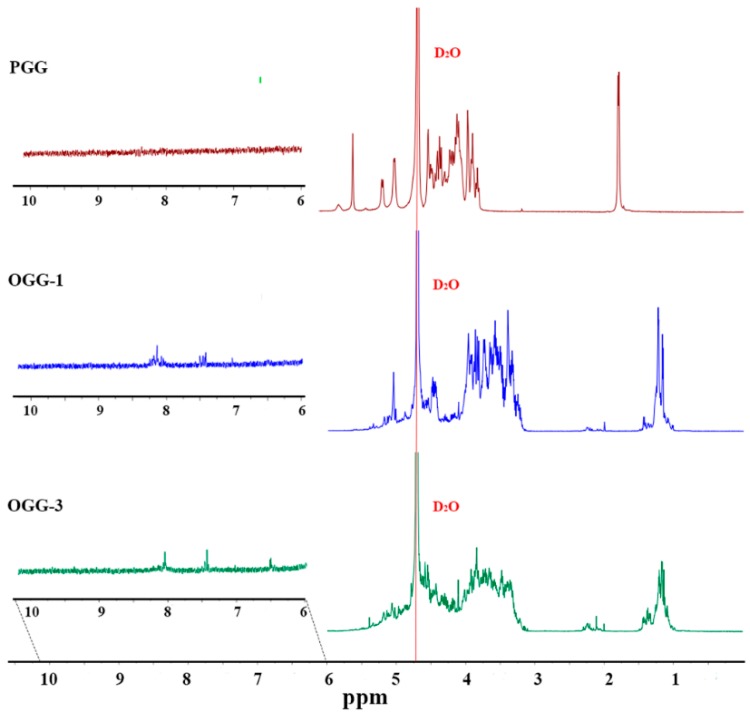
The ^1^H-NMR spectra of gellan gum (PGG) and oxidized gellan gum (OGG-1 and OGG-3).

The ^1^H-NMR of the low acyl gellan gum, OGG-1 and OGG-3 were tested and compared. Samples 1 and 3 have been demonstrated with different oxidation levels. The ^1^H-NMR spectrum is shown in Figure 2. The obtained spectra of gellan gum showed characteristic peaks corresponding to -CH_3_ of rhamnose (*δ* 1.20–1.4 ppm), -CH- of glucose, glucuronic acid, and rhamnose (*δ* 3.8–4.9 ppm), and -CH- of glycosidic bonds in sugars (*δ* 5.0–5.6 ppm). The peaks at 5.6, 5.4 and 5.1 ppm were assigned to the H of C1 with glucoside bonds of the rhamnose, glucuronic acid, and glucose, respectively [23]. Because there is a dissolving problem of gellan gum in cold water, the NMR spectrum of gellan gum was tested at 80 °C. The chemical shifts of OGG-1 and OGG-3 shifted to lower *δ* values at a low temperature. 

It was seen that the original single peak of -CH_3_ on the rhamnose structure (around *δ* 1.2 for the OGGs) split into the multiple peaks in the OGGs. Peaks corresponding to the -CH- of glycosidic bonds, especially at *δ* value of 5.1 (corresponding to the glycosidic bond of rhamnose in the OGGs), became smaller than these in the pure gellan gum. Moreover, these changes became more obvious as the oxidation level increased. The oxidation of the glucoside bond in the rhamnose unit made the proton environments of the -CH_3_ group become different which led to the split of the original peaks. These results demonstrated that the partial cleavage of the glucosidic linkages occured in the oxidation process of the gellan gum by H_2_O_2_. Some small and new peaks appeared in the 2.0–2.3 ppm and 6.5–7.5 ppm regions, which did not belong to the normal sugar structures [23]. It is interesting to find that small peaks in these regions could also be found in the H-NMR spectrum of oxidized gellan gum using NaIO_4_ as oxidizing agents by Gong et al. [12]. We supposed that some acetylate or ester groups were introduced from the oxidation process [24]. However, it was hard to explain these molecular structures. It was also strange that no peaks, even small, were found at the region larger than 8.05 ppm which belonged to the terminal aldehyde group [12]. It suggested that there may not be any terminal aldehyde groups in the OGGs obtained via Fenton-type oxidation, and the carbonyl contents determined may belong to the ketone or carboxyl groups. The results were in accordance with the FTIR results that carboxyl groups are favorably formed in the Fenton-type oxidation of polysaccharides. 

### 3.2. Oxidation Mechanisms

**Figure 3:**
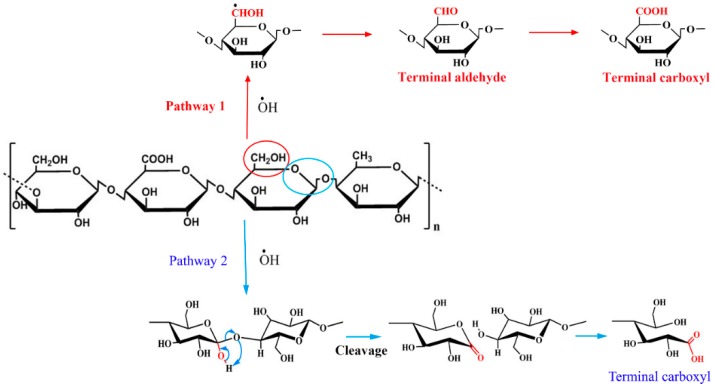
Pathways for gellan gum oxidation in H_2_O_2_ in the presence of ions.

Many studies have used H_2_O_2_ as an oxidant and copper sulfate as a catalyst to oxidize polysaccharides with a mechanism belonging to the scope of Fenton chemistry. [16,17,18,25] In the presence of a metal catalyst, the mechanisms of polysaccharides oxidation by H_2_O_2_ are very complex [26,27]. According to the Fenton reaction, H_2_O_2_ decomposes rapidly and forms hydroxyl radicals in the presence of transition metal. These hydroxyl radicals are very powerful oxidizing species which can oxidize polysaccharides rapidly. The low acyl gellan gum structure consists of monosaccharides *β*-D-glucose, *α*-L-rhamnose and *β*-D-glucuronic acid in molar ratios of 2:1:1 linked together by glycosidic bonds. According to references, we supposed that there might be two possible oxidative pathways as shown in Figure 3. 

Pathway 1 is proposed via the oxidation of hydroxyl groups such as C6 hydroxyl to carbonyl groups and then to carboxyl groups [26]. As shown in Table 1, the major functional group produced was carboxyl which progressively increased with the H_2_O_2_ amount while only little carbonyl groups were formed. The results agreed with earlier studies that the oxidation of polysaccharides favors the formation of carboxyl groups [16,17,28]. It was suggested that once the carbonyl groups were formed, they would be rapidly converted to carboxyl groups [28]. Therefore, only a small amount of carbonyl groups were accumulated as shown in this study. Parallel reaction paths in which carboxyl groups are formed by the oxidation of glycoside bonds as shown in pathway 2 were also reported [29]. As shown in the figure, the attacking of the C1 position in sugar by hydroxyl radicals leads to the cleavage of glycoside bonds of polysaccharides and forms acidic functionality groups. Recently, this pathway was reported as the main molecular mechanism of polysaccharide oxidative cleavage via a Fenton-type reaction in the presence of a copper enzyme [30]. This oxidative cleavage would lead to varied small linear polysaccharides with functional carboxyl groups such as a glucuronic acid group. It is suggested that pathway 2 is dominated in the oxidation process.

### 3.3. Gelation Properties

#### Gelling Properties of the Oxidized Gellan Gums

**Figure 4:**
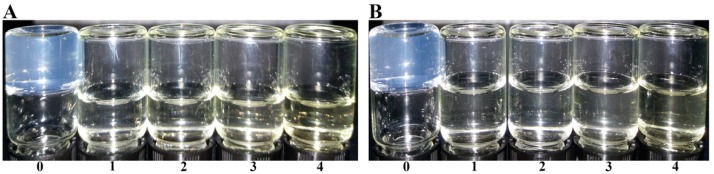
Gelation of pure gellan gum (PGG) and oxidized gellan gum (OGG) in different ionic conditions: (**A**) Ca^2+^, (**B**) Na^+^. The samples are (0) PGG, (1) OGG-1, (2) OGG-2, (3) OGG-3, (4) OGG-4.

**Figure 5:**
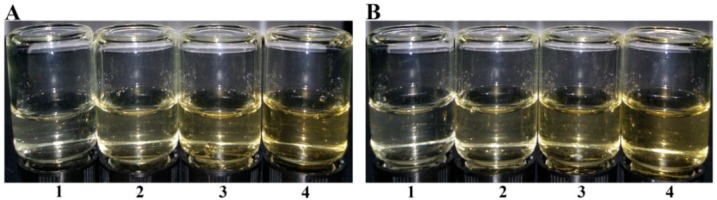
Gel conditions with different OGG-3 content: (**A**) Ca^2+^, (**B**) Na^+^. The contents are (1) 0.4%, (2) 0.8%, (3) 1.0%, (4) 1.2%.

The low acyl gellan gum has a property to form a gel in the presence of metal ions such as Na^+^ and Ca^2+^. Therefore, the gel formation properties of the OGGs were tested with the addition of ions as shown in Figure 4. However, all the OGGs could no longer form a gel even with the quite high concentration (up to 1.2%, Figure 5). The gelation of gellan gum is considered as the formation of double-helical junction zones which is associated and stabilized by cations and water molecules [31]. The oxidation of hydroxyl groups and the cleavage of glycoside bonds may strongly break the intramolecular hydrogen bonds, which leads to poor gelation stability. 

Meanwhile, it was found that the oxidation of the gellan gum could significantly improve its cold water solubility, which could not be found in the gellan gum. Therefore, we assumed that the oxidation results were in more disorder in structure conformations and the loss of crystallinity, which made the OGGs easily dissolved in water but with poor gelling ability. However, Gong et al. [12] used NaIO_4_ to oxidatively cleave the low acyl gellan gum backbones which could still maintain its gelling ability. This difference may result from the different reaction reagent used and oxidation positions in the molecular structures. 

### 3.4. Antimicrobial Activities and Food Coating Applications

#### 3.4.1. Antimicrobial Activities of the Oxidized Gellan Gums

**Figure 6:**
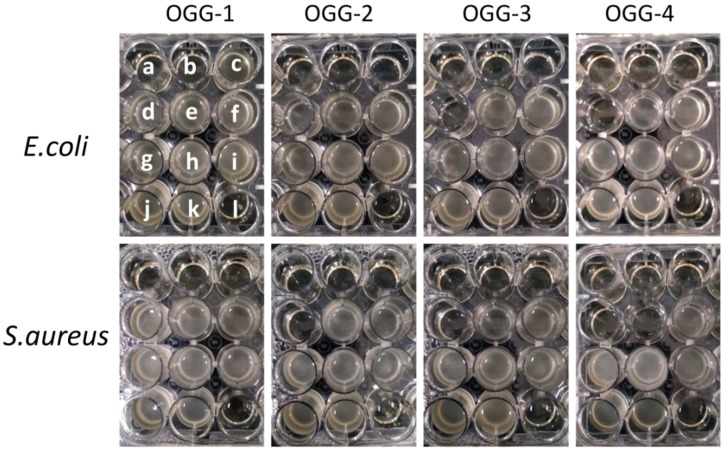
Photographs of bacterial colonies grown in NB (nutrient broth) containing different contents of oxidized gellan gum (OGG): (a) 80 mg/mL, (b) 40 mg/mL, (c) 20 mg/mL, (d) 10 mg/mL, (e) 5 mg/mL, (f) 2.5 mg/mL, (g) 1.25 mg/mL, (h) 0.63 mg/mL, (i) 0.32 mg/mL, (j) 0.16 mg/mL, (k) 0 mg/mL, (l) NB medium.

The MIC of the OGGs against *E. coli* and *S. aureus* were measured to evaluate the antimicrobial activities. Figure 6 shows the photographs of *S. aureus* and *E. coli* colonies grown in NB broths containing different contents of the OGGs. The values of MIC are shown in Table 2. The OGGs present a broad-spectrum antibacterial activity, which is improved with an increasing oxidation level. The OGG-4 presents the lowest MIC, which was similar to the values of oxidized schizophyllan [32]. It is proposed that the antibacterial activity of the OGGs is conferred by the new generated carboxyl groups. Abaev et al. [33] reported that the number of carboxylic groups attached to oxidized cellulosic materials was directly proportional to their antibacterial activity against *S.aureus*. Recently, Zi et al. [34] studied the effects of aldehyde and carboxyl groups on the antibacterial activity of oxidized amylose. They concluded that the antibacterial activity of dicarboxyl-amyloses was due to the acidic pH effect produced by the carboxyl groups [29].

**Figure 7:**
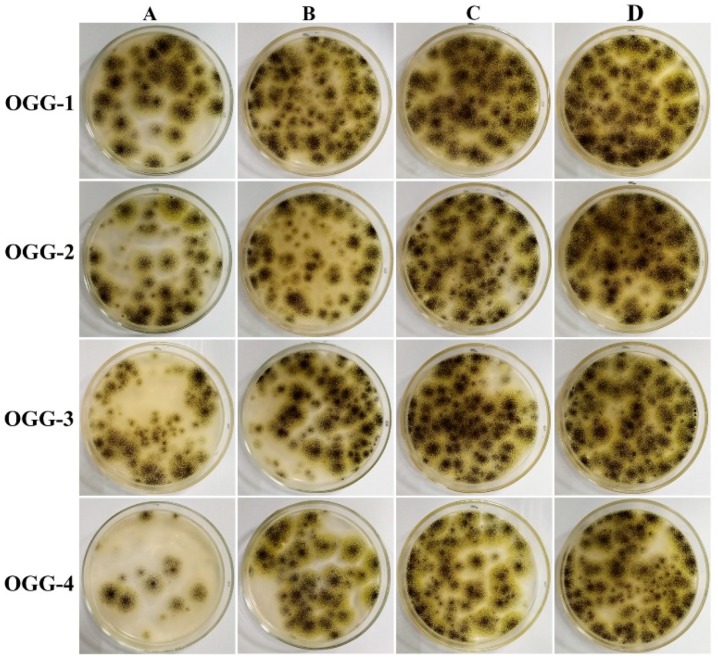
Typical photographs of *A. niger* colonies grown on potato dextrose agar plates: (**A**) 40 mg/mL, (**B**) 20 mg/mL, (**C**) 10 mg/mL, (**D**) 5 mg/mL.

The antifungal activities of the OGGs were tested against *A. niger* colonies. The typical photographs of *A. niger* colonies grown on PDA plates are shown in Figure 7. The results showed that the growth of *A. niger* could be inhibited by a certain amount of the OGGs. At the same oxidation level (line in the figure), the number of *A. niger* colonies decreased steadily with an increasing concentration of the OGGs. Moreover, the higher the oxidation level at the same concentration (row in the figure), the less amount of *A. niger* colonies were found on the plate. Among them, the lowest number of *A. niger* colonies was in a concentration of 40 mg/mL of the OGG-4. These results showed that the OGGs had antifungal activity. Again, we propose that the antifungal activities of the OGGs are attributed to their carboxyl groups. 

The results above showed that the OGGs could affect a relatively broad spectrum of microorganisms, although the inhibitory concentrations were large. It is known that a pH of 4.4–9.0 is the limiting range and more acidic conditions can be lethal to most organisms. The OGGs with sufficient carboxyl groups can increase the proton concentration thereby decreasing the environmental pH of microorganisms. This will alter the permeability and integrity of the microbial cell membranes, and also exert some disturbance in the nutrients transport, which causes the inactivation and death of microbes [17,34,35]. This antimicrobial action does not act in a mechanism-specific manner which will lead to antibiotic-resistant strains, as many antibiotics. 

#### 3.4.2. Effect of the Oxidized Gellan Gums on Fruit Preservation as Antimicrobial Coatings

**Figure 8:**
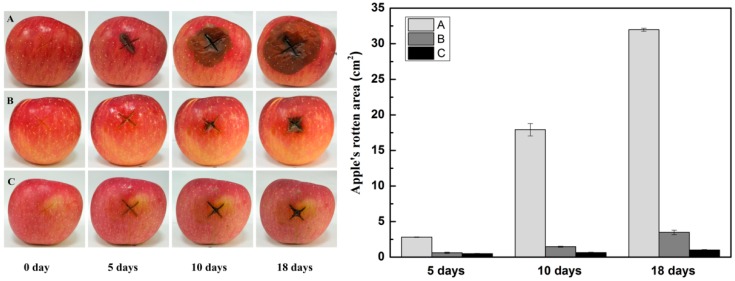
The appearance of apples after treatment by different solutions for different days: (**A**) 0 mg/mL OGG-4 solution; (**B**) 10 mg/mL OGG-4 solution; (**C**) 20 mg/mL OGG-4 solution.

The oxidized polysaccharides such as the OGGs prepared by green methods can be used as antimicrobial edible coatings for food preservation [18]. As an example, the OGGs were used as active coating materials in the preservation of apples [18]. Figure 8 shows the appearance of apples treated with different solutions of the OGG-4 after storage for a period of time. The decayed areas of the apples are shown on the right side of the figure. The pH was determined as 6.3, 3.3, and 3.1 for the control, 10 mg/mL OGG-4 solution, and 20 mg/mL OGG-4 solution, respectively. It can be seen that control one began to decay in 5 days. When the solution contained OGGs, the apple began to decay in 10 days. As the concentration of the OGGs increased, the rate of rot became slower. The results clearly showed that solutions containing the OGGs could effectively extend apple storage time due to their antibacterial and antifungal activities [18]. Therefore, the OGGs have potential applications as coating materials for food preservation. 

## 4. Conclusions

The oxidized gellan gums with different oxidation levels were successfully prepared by hydrogen peroxide and the copper sulfate redox system. The infrared spectrum and ^1^H-NMR spectroscopy analysis demonstrated that the carboxyl group had been successfully introduced into the gum structure. The oxidation mechanisms were discussed and the cleavage of glycoside bonds might occur. The OGGs lost the abilities of gelation even in the presence of ions. Oxidation gives the OGGs a broad-spectrum antimicrobial activity against bacterials (*Escherichia coli* and *Staphylococcus aureus*) and fungus (*A. niger*), and the activity increases with an increase in the oxidation level. The OGGs are applied as coating materials and can extend the storage time of apples. The study shows that new gellan gum derivatives by Fenton type oxidation have potential applications as antimicrobial coating materials in the food industry. 

## Figures and Tables

**Table 1:** Carboxyl content and carbonyl content of oxidized gellan gum.

Sample	Carboxyl Content (%)	Carbonyl Content (%)
PGG	-	-
OGG-1	6.11 ± 0.05	0.36 ± 0.01
OGG-2	7.96 ± 0.04	0.44 ± 0.03
OGG-3	11.82 ± 0.01	0.75 ± 0.04
OGG-4	14.51 ± 0.03	0.87 ± 0.02

PGG: Pure gellan gum; OGG: Oxidized gellan gum.

**Table 2:** Minimum inhibitory concentration (MIC) of oxidized gellan gum with different oxidation levels on *E. coli* and *S. aureus*.

Bacteria	Sample	MIC (mg/mL)
*E. coli*	PGG	--
	OGG-1	40
	OGG-2	20
	OGG-3	20
	OGG-4	10
*S. aureus*	PGG	--
	OGG-1	20
	OGG-2	20
	OGG-3	10
	OGG-4	5
